# Autonomous Fingerprinting and Large Experimental Data Set for Visible Light Positioning

**DOI:** 10.3390/s21093256

**Published:** 2021-05-08

**Authors:** Tyrel Glass, Fakhrul Alam, Mathew Legg, Frazer Noble

**Affiliations:** Department of Mechanical & Electrical Engineering, SF&AT, Massey University, Auckland 0632, New Zealand; t.glass@massey.ac.nz (T.G.); m.legg@massey.ac.nz (M.L.); f.k.noble@massey.ac.nz (F.N.)

**Keywords:** fingerprint, Indoor Localization, Indoor Positioning Systems (IPS), Virtual Reality (VR), ground truth, Visible Light Positioning

## Abstract

This paper presents an autonomous method of collecting data for Visible Light Positioning (VLP) and a comprehensive investigation of VLP using a large set of experimental data. Received Signal Strength (RSS) data are efficiently collected using a novel method that utilizes consumer grade Virtual Reality (VR) tracking for accurate ground truth recording. An investigation into the accuracy of the ground truth system showed median and 90th percentile errors of 4.24 and 7.35 mm, respectively. Co-locating a VR tracker with a photodiode-equipped VLP receiver on a mobile robotic platform allows fingerprinting on a scale and accuracy that has not been possible with traditional manual collection methods. RSS data at 7344 locations within a 6.3 × 6.9 m test space fitted with 11 VLP luminaires is collected and has been made available for researchers. The quality and the volume of the data allow for a robust study of Machine Learning (ML)- and channel model-based positioning utilizing visible light. Among the ML-based techniques, ridge regression is found to be the most accurate, outperforming Weighted k Nearest Neighbor, Multilayer Perceptron, and random forest, among others. Model-based positioning is more accurate than ML techniques when a small data set is available for calibration and training. However, if a large data set is available for training, ML-based positioning outperforms its model-based counterparts in terms of localization accuracy.

## 1. Introduction

Localization and navigation systems have been widely used since the development of Global Positioning System (GPS) [1]. However, GPS struggles to operate reliably indoors due to signal degradation and multipath propagation [2]. A large number of potential applications such as smart guidance in large facilities, e.g., hospitals and shopping malls, ambient assisted living in smart homes, and asset tracking have led researchers to develop Indoor Positioning System (IPS) using a wide variety of techniques including Infrared (IR) [3], Wi-Fi [4], Bluetooth [5], Zigbee [6], ultra-wideband (UWB) [7], acoustics [8], and computer vision systems [9].

The rapid adoption of the Light Emitting Diode (LED) for illumination and the advancement of Visible Light Communication (VLC) [10] technology have made Visible Light Positioning (VLP) [11] a promising option for IPS. VLP falls into two broad categories: camera-based [12] and photodiode-based [13]. The lower price and power costs of photodiodes make them preferable for certain applications such as asset tracking. While several signal characteristics like Angle of Arrival (AOA), Time of Arrival (TOA), Time Difference of Arrival (TDOA), etc. have been reported in the literature, Received Signal Strength (RSS) is most widely utilized for photodiode-based VLP [14]. RSS-based positioning can be achieved with cost-effective hardware without requiring any synchronized infrastructure.

RSS-based VLP can be broadly categorized into model-based [15] or fingerprinting approaches [16]. Model-based approaches develop calibrated signal attenuation–distance relationships that are used to estimate the range of the target from multiple luminaires whose locations are known a priori. Once three or more ranges are available, the target can be localized. Fingerprint techniques, on the other hand, capture a labeled offline data set of locations and corresponding RSS of multiple luminaires and use this to build a machine learning algorithm capable of predicting online locations. For both approaches, there is a need for a large number of experimental measurements of RSS and the positions at which they were taken with high confidence in accuracy. This data is used to calibrate the model, train the algorithms (e.g., regressors) as well as to evaluate the accuracy of the localization system.

Collecting accurate ground truth data is a major challenge for VLP and indoor positioning research. Current techniques are labor-intensive and restrict the number of data that can be collected. Generally, data collection is accomplished by placing a receiver on a series of pre-marked reference locations and manually recording the position and the corresponding RSS. This method requires a substantial time investment for both measuring the reference locations as well as the numerous repositioning of the receiver during data collection. The painstaking fashion of the method can also introduce error as user fatigue develops over long sessions of data collection. This has resulted in VLP research being conducted with data sets of limited size. Table 1 presents the data collection sizes for recent VLP experiments. Most of the reported testbeds are much smaller than a typical real-world environment, which makes it difficult to assess how the findings would translate into operational performance. Furthermore, the largest recorded data set has only 693 recorded locations [17], which is an order of magnitude lower than that presented in this paper. Furthermore, most of the works do not discuss the ground truth recording methodology when, in most cases, reporting mean localization errors of 50 mm or lower. This also casts doubt on the accuracy of such reported measurements.

The lack of large data sets has led to researchers collecting small data sets and then using interpolation [23] and regeneration [17] to produce larger fingerprints. However, due to non-ideal signal characteristics such as multipath propagation, such methods are less accurate. When training Machine Learning (ML) models, regularization has been used to accommodate small training data sets [20,21]. However, due to the lack of sufficient testing points, it is difficult to assess the generality of the models to data not encountered, and the small volume of data used dramatically increases the likelihood of overfit. Using small data sets reduces the accuracy scores for both fingerprinting and model-based methods as there are not enough testing points to provide a robust sample. Furthermore, the difficulty in collecting data severely limits the effectiveness of fingerprinting systems in changing environments as a new data set would be required after any part of the environment is altered.

A system that can automatically capture the ground truth position of a VLP receiver (often termed as “tag”) accurately would resolve the issues present with current data collection methods. High accuracy commercial systems, such as motion capture and SLAM exist; however, they are prohibitively expensive, and the sensors can be bulky. Recently, lower-cost systems using ultrasonic localization have been used [24]. However, such systems have a mean accuracy of ~20 mm [25], which is several times worse than the Vive. This paper outlines the use of the HTC Vive (https://www.vive.com/nz/accessory/vive-tracker/ (accessed on March 30 2021)) tracking platform, specifically the lighthouse base stations and tracker, for ground truth recording. The Vive system is an order of magnitude more accurate than VLP systems, allowing it to reliably evaluate the accuracy of the VLP system under investigation. The Vive system is used along with a Xiaomi Roborock s5 (https://us.roborock.com/pages/roborock-s5 (accessed on 30 March 2021)) robotic vacuum that automates the repositioning of the target around the test area, making the entire data collection system free from user involvement once the setup is complete. The paper offers the following contributions:

1. It presents a new technique for automating the collection of large VLP data sets with high positioning accuracy and wide area coverage. This allows high accuracy calibration and validation of VLP systems. This is the first time such a system has been reported in the literature.
2. The implementational details of the ground truth recording system are provided. This will enable other researchers to develop their own systems to collect VLP data over an arbitrarily large area. We also discuss key issues like mitigating the Vive’s IR interference and the robotic platform’s Electromagnetic Interference (EMI) on the VLP system. Experimental study demonstrates the consistency and the accuracy of the Vive’s positioning performance.
3. A large data set containing visible light RSS from 11 luminaires at 7344 test locations over a 43.5 m^2^ area is constructed and made available online for other researchers to utilize in their own research. To the best of the authors’ knowledge, this is the largest collected data set for VLP and is an order of magnitude bigger than previously reported ones.
4. The data set is used to provide example evaluations of common machine learning-based positioning. We demonstrate that a large data set is necessary not only for training models, but also for robust performance comparisons. For large data sets, it is found that ridge regression outperforms other machine learning techniques in terms of localization accuracy. The Lambertian RSS-distance channel model is calibrated for ranging that enables localization using linear-least-squares- and iterative-spring-relaxation-based lateration. Such model-based positioning is shown to be well suited for a small data set, with the ML-based positioning being superior when a large data set is available.

The rest of the paper is organized as follows. Section 2 describes the components used for data collection: the VLP system, the Vive tracking including an analysis of ground truth performance and co-ordinate transformation, and use of the robotic vacuum platform. Section 3 describes how the experiments were conducted and compares the data collected with theoretical models. Section 4 details the performance of some common ML-based localization algorithms trained on the collected data, and Section 5 does the same with two channel-model-based methods. Section 6 highlights the need for large data sets for both training and testing. Section 7 concludes the paper with suggestions for future work.

## 2. System Overview

### 2.1. Experimental Setup

**Figure 1:**
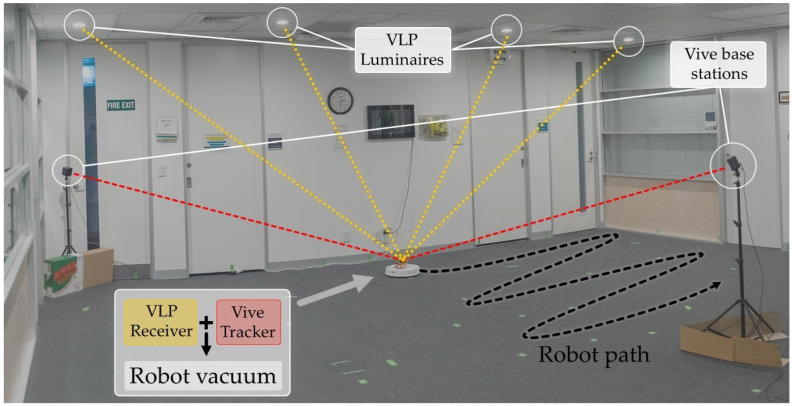
Office lobby used for the experiment. Two Vive base stations provide accurate positioning for the Vive tracker, which is co-located with the VLP receiver on a mobile robot vacuum.

The test environment consisted of a 6.5 × 7 × 2.4 m office lobby that was cleared of furniture (see Figure 1). There are 11 bespoke luminaires spaced as evenly as possible within the constraints imposed by the existing lighting and air conditioning installations. A pillar is present in the room that obstructs the light from several luminaires in specific locations.

### 2.2. VLP System

**Figure 2:**
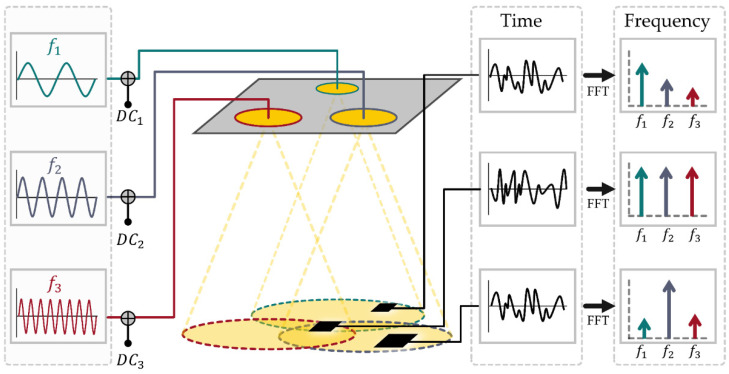
FDM scheme demonstrated for three luminaires. Each luminaire is driven by a DC signal overlaid with a sinusoid of unique frequency. A photodiode detector receives a signal that is the sum of multiple luminaire’s outputs scaled by distance. This signal is then converted to an FFT, and the component at each luminaire’s modulating frequency is used as the RSS.

**Figure 3:**
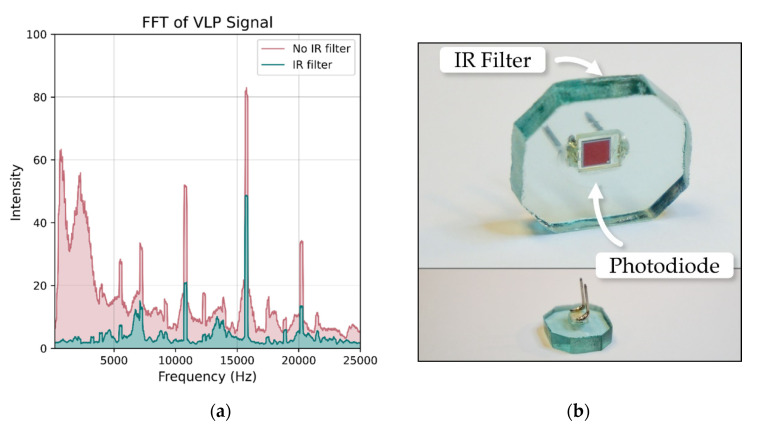
The IR signals from the Vive base stations caused interference with the VLP receivers as shown in (**a**). The Vive signal added substantial noise at frequencies lower than 4 kHz and altered the intensities of the luminaire peaks. To prevent interference, an IR-rejecting photodiode was used and then placed behind IR-rejecting glass (**b**). The combination of the two eliminated the interference.

The details of the VLP system employed in this work can be found in reference [13]. The 11 luminaires used in the experimental setup were distinguished from each other using frequency division multiplexing (see Figure 2), with custom driver boards inserting an unmodulated sine wave between 2 to 22 kHz. The receiver tag comprises a photodiode feeding into a trans-impedance amplifier with filtering to remove ambient light fluctuation and 100 Hz power line flicker. It was found that the IR signals emitted from the Vive lighthouses interfered with the VLP system (Figure 3a). To overcome this, an IR-rejecting photodiode (https://dammedia.osram.info/media/resource/hires/osram-dam-5467146/SFH%202440_EN.pdf (accessed on 30 March 2021)) was used and placed behind a section of IR blocking glass (Figure 3b).

The received signal at the output of the amplifier sampled at a rate of 50 kHz by an STM32 (https://www.st.com/en/microcontrollers-microprocessors/stm32f103cb.html (accessed on 30 March 2021)) microcontroller and data sent to an ESP8266 (https://www.espressif.com/en/products/socs/esp8266 (accessed on 30 March 2021)) Wi-Fi chip that hosts an HTTP server. A computer connected to the local network can obtain a new reading using a simple HTTP GET request. Demultiplexing and RSS extraction is performed using Fast Fourier Transform (FFT). The power of the resulting spectra at each of the 11 multiplexing frequencies are used as a measure of the RSS for the corresponding luminaire.

### 2.3. HTC Vive Tracking for Accurate Ground Truth

Accurate calibration and benchmarking of the performance VLP localization methods require precise knowledge of the locations where VLP data measurements are made. To achieve this, the HTC Vibe tracking system [26] was used. This system enables high-precision positioning for Virtual Reality (VR). Its low cost and accurate tracking performance have led to interest in its use for various research [27,28,29,30,31]. The basic tracking system consists of two or more fixed “Lighthouse” base stations and a mobile Tracker. The system has been reported to provide tracking accuracy of about 5.8 mm [31] and therefore seems suitable for determining the ground truth position during an experimental data collection for VLP. Co-locating the Vive tracker with a VLP receiver eliminates the need for painstaking manual measurements.

The tracker provides 6 DoF (degrees of freedom) localization ($\hat{X},\hat{Y},\hat{Z},\gamma ,\beta ,\alpha$) and uses Bluetooth to communicate to a wireless dongle connected to a host PC running SteamVR (https://store.steampowered.com/steamvr (accessed on 30 March 2021)). The pyopenvr [32] library provides Python bindings for the OpenVR virtual reality SDK [33] that accesses SteamVR, allowing for scripting control of the tracking system. The Triad OpenVR wrapper [34] simplifies the interface, providing a simple Python function to request the tracker’s current position.

**Figure 4:**
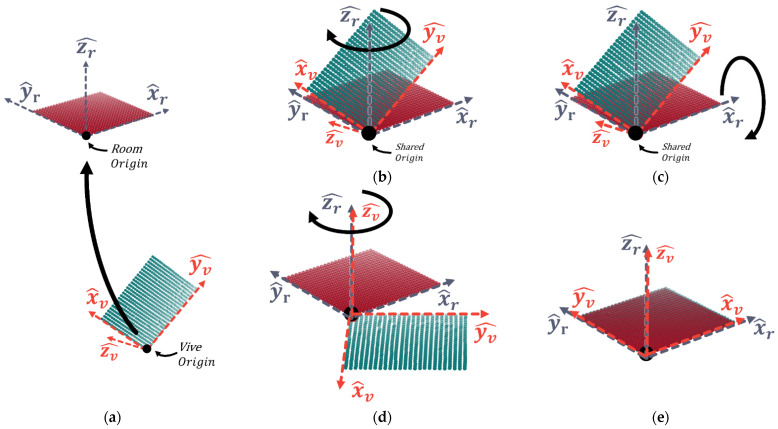
Using Z-X-Z Euler angles to derive an initial alignment between the vive co-ordinate reference frame $\left({\hat{x}}_{v},{\hat{y}}_{v},{\hat{z}}_{v}\right)$ and the room co-ordinate frame $\left({\hat{x}}_{r},{\hat{y}}_{r},{\hat{z}}_{r}\right)$. A translation aligns the origins (**a**); then, ${\hat{z}}_{v}$ is aligned with the ${\hat{z}}_{r},{\hat{y}}_{r}$ plane via rotation around ${\hat{z}}_{r}$ (**b**). This allows a rotation around ${\hat{x}}_{r}$ to align ${\hat{z}}_{r}$ with ${\hat{z}}_{v}$ (**c**). Finally, another rotation around $\hat{z_{r}}$ (**d**) completes the alignment (**e**).

The tracker’s location is provided with respect to a local co-ordinate system centered at the first base station with axes aligned along its orientation, which is tilted during setup (Figure 4a). This necessitates a transformation to map between the reference co-ordinate frames. Deriving this transform involves placing the tracker at known locations within the test environment and building a set of points in each reference frame with the same location. The mapping from the Vive to the room co-ordinates can then be derived and applied to any subsequently recorded points.

In theory, only three non-colinear reference points are required to define the transform. However, any errors in these points would be propagated to the final transform, and the use of multiple points reduces the likelihood of error. To avoid an over-defined solution when using multiple points, the Iterative Closest Point (ICP) algorithm [35], a point set registration technique for determining the spatial transformation that aligns two sets of points, is used to determine the final transform. The algorithm first assigns a correspondence from the target set to the point set using a nearest-neighbor approach. A transformation is then derived using a translation followed by rotation to attempt to align the matched points. The process is repeated with both the transformation and correspondence, improving at each iteration until the point-to-point distance is minimized, or a maximum number of iterations is reached.

To ensure convergence of the ICP, a rough initial alignment is required so that the point-to-point correspondence settles on the correct mapping. This alignment is derived using Euler’s rotation theorem [35] applied to points that roughly align with the three co-ordinate unit vectors. First, the origins of the two reference frames are aligned by applying a translation (see Equation (1) and Figure 4a):

(1) $$T=\left[\begin{array}{c} -local\;origin\prime s\;\left(x\right)\\ -local\;origin\prime s\;\left(y\right)\\ -local\;origin^{\prime }s\;\left(z\right) \end{array}\right]$$

Next are three rotations that follow the form of a $\hat{Z}$-$\hat{Y}$-$\hat{Z}$ fixed angle set [36].

(2) $$\;R_{z}\;\left(\gamma \right)=\left[\begin{array}{ccc} cos\;\gamma & -\sin\gamma & 0\\ \sin\gamma & \cos\gamma & 0\\ 0 & 0 & 1 \end{array}\right]$$

(3) $$R_{x}\;\left(\beta \right)=\left[\begin{array}{ccc} cos\;\beta & 0 & sin\;\beta \\ 0 & 1 & 0\\ -sin\;\beta & 0 & cos\;\beta \end{array}\right]$$

(4) $$R_{z}\;\left(\alpha \right)=\left[\begin{array}{ccc} cos\;\alpha & -\sin\alpha & 0\\ \sin\alpha & \cos\alpha & 0\\ 0 & 0 & 1 \end{array}\right]$$

where *γ*, *β*, and α are the rotation angles around $\hat{Z}$, $\hat{Y}$, and $\hat{Z}$ axes, respectively. *γ*, *β,* and *α* approximations are derived by using dot and cross products between the points roughly aligning with the unit vectors in each co-ordinate system. All the transformation matrices then can be used to perform transformation of a [3×N] matrix of array of N measurement point co-ordinates $X_{v}$ from the Vive’s co-ordinate system into the room’s co-ordinate system using

(5) $$X_{room\;}=(R_{z}\cdot R_{x}\cdot R_{z\;})\;X_{v}+T$$

Figure 4 shows the step-by-step transformation. After the rough alignment between calibration points, the library open3d (http://www.open3d.org/ (accessed on 30 March 2021)) is used to implement the ICP algorithm deriving the transformation matrix from the Vive co-ordinate frame to the rooms. This transformation matrix needs to be derived each time a new experimental setup is used. In the next sub-section, we outline an analysis of the number of calibration points required to accurately determine the transformation.

### 2.4. Vive Performance Analysis

Two sets of experiments were done to verify the accuracy of the Vive positioning system. Firstly, the system was tested for ‘jitter’, the amount of variation the system reported for a stationary object. Secondly, the tracking accuracy was investigated using a Computer Numerical Control (CNC) platform with a resolution of 0.025 mm.

#### 2.4.1. Consistency of Vive’s Position Measurement

To investigate the system’s consistency, the two Vive base stations were placed 3 m apart, and the tracker was kept immobile on a stationary surface in the view of both stations. Two data collection runs were repeated: A and B. For both runs, 100 measurements were taken at intervals of 1.5 s. Runs A and B were conducted with the tracker at different locations and orientations to determine if these factors affected measurement consistency.

Table 2 shows metrics for the difference in the Euclidean distance between the estimated position and mean for the 100 readings. The median, 90th percentile, and max variations were below 0.15, 0.35, and 0.65 mm, respectively, for both runs. These results indicate the positional variation is small enough to avoid needing multiple ground truth readings (with subsequent averaging) at any test location. These results are in agreement with previously reported work [31].

**Figure 5:**
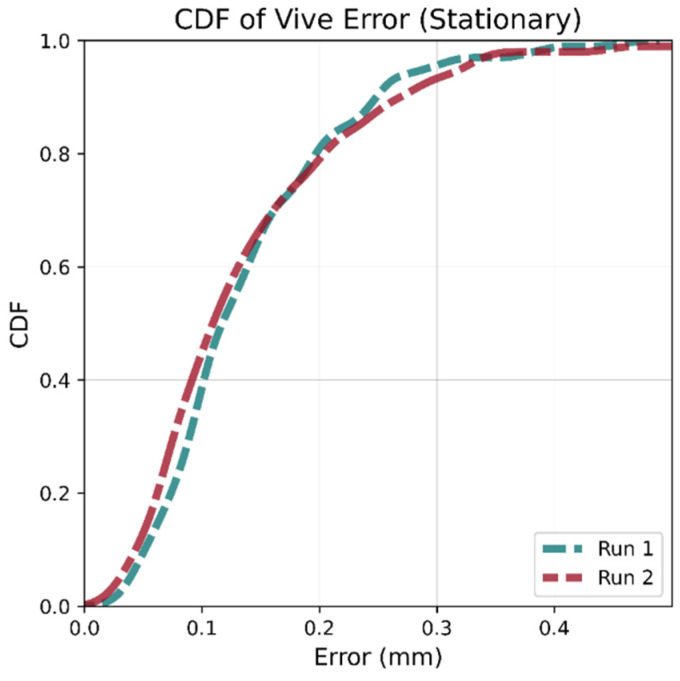
CDF for Vive jitter at different locations and orientations. The two runs are very similar indicating that there is no spatial dependence.

It can also be seen in Figure 5 that the Cumulative Distribution Function (CDF) of the error distance for all one hundred points for both runs are very similar despite having different orientations and being placed in different locations, suggesting that the system has a low systematic error.

#### 2.4.2. Accuracy of the Vive System

**Figure 6:**
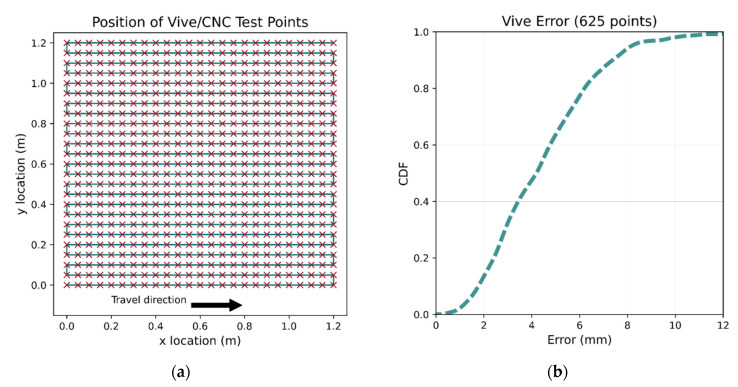
Determination of the Vive accuracy. The tracker was moved to 625 locations (**a**) by the CNC machine. The high accuracy of the CNC allows the Vive errors to be accurately calculated. The error CDF is shown in (**b**); median error is less than 4.5 mm.

To investigate the system’s accuracy, the performance of the VR system was tested using a custom-made 2D CNC machine with an accuracy of 0.025 mm. The CNC machine’s tool’s position can be set by providing the desired x,y co-ordinates. Mounting the Vive tracker to the tool allows its true position to be accurately determined, which can then be compared with the Vive positional outputs. A total of 625 tracker position measurements were taken at 50 mm intervals within a 1.2 × 1.2 m space shown in Figure 6a, the orientation of the tracker was kept constant. Rough alignment followed by ICP was applied to the Vive’s output to align the two co-ordinate reference frames, with the ICP algorithm using all data points to derive the transformation. The error CDF for the CNC points is shown in Figure 6b.

#### 2.4.3. Number of Calibration Points Needed

The results presented in the previous sub-section demonstrate that the ICP algorithm can map the Vive’s position co-ordinates to the room’s reference frame with high accuracy. However, the ICP used all 625 points when finding the best transformation. Due to the impracticality of utilizing such a large number of calibration points, an investigation into the accuracy of the transformation using a limited number of points was undertaken, first using points at random, then using carefully chosen locations.

To evaluate the accuracy of ICP when using p number of points, a transformation matrix is computed using the p points and then applied to all 625 points. The accuracy is then computed as the mean distance from each transformed Vive point to the ground truth position provided by the CNC. Starting with p=3, (the minimum number of points required to derive the transformation) and increasing the number of points provides a way to determine the number required.

**Figure 7:**
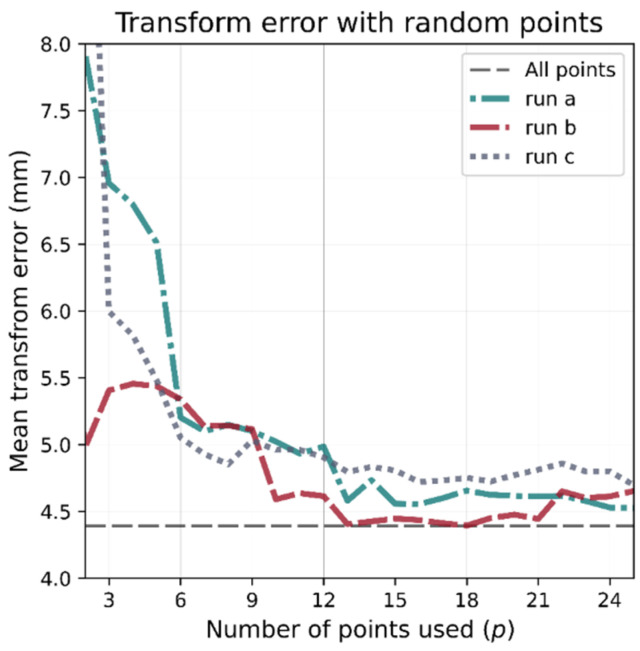
Dependence of transform accuracy on the number of calibration points. As more points are used, accuracy improves. The dashed grey line represents the ideal transform calculated using all 625 points. Using randomly chosen calibration points produces inconsistent results.

Figure 7 shows the mean accuracy error vs. the number of calibration points when the points are chosen at random. Three different runs are plotted, each one using a different random shuffling of the 625 points. As p is increased, the accuracy of the transformation improves; however, the random selection of points produces inconsistent results. Since this work required 2D ground truth, the Z-axis was nullified when comparing the transformations, future work involving 3D positioning will need to re-validate the optimum number of reference points needed.

**Figure 8:**
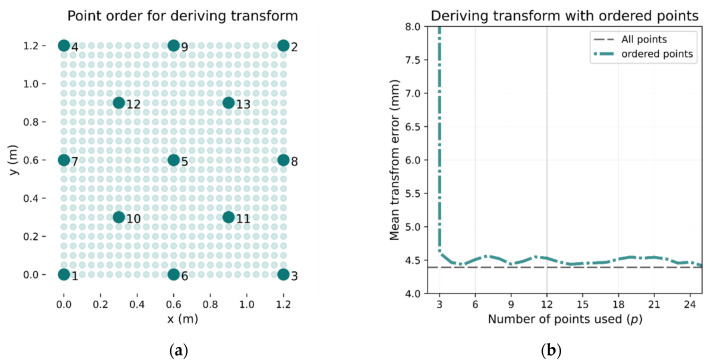
Selecting calibration points to maximize area covered. If points are chosen by following the pattern in (**a**), then the transform accuracy converges much faster, as can be seen in (**b**). With only 3 calibration points, the mean error is below 5 mm (shown in (**b**)).

If, rather than selecting the calibration points randomly, each new point is chosen to be as far as possible from any existing calibration point, fewer points are needed. An example of such point selection is shown in Figure 8a. If the initial point is at the bottom right (point 1), the next point selected will be point 2 (top right) as this is the furthest away. Subsequent points are then chosen to be as far away from any other calibration point as possible. When using this method, the transformation error converges close to the lowest overall error with fewer calibration points as demonstrated in Figure 8b. It can be seen that five calibration points result in an error that remains below 5 mm.

### 2.5. Roborock S50 Robotic Platform

**Figure 9:**
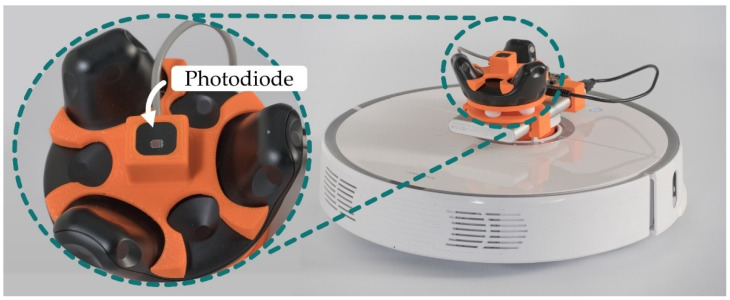
The Vive tracker was mounted to the top of the Roborock 55 with care taken to avoid blocking the LIDAR. The VLP tag was mounted in the center of the tracker without blocking the Vive’s photodiodes. Both the Vive tracker and the VLP tag were powered using a USB power bank.

To eliminate the need for manual repositioning of the target node, the Roborock S5 robotic vacuum platform was used. Both the Vive Tracker and VLP receiver were mounted to the top of the vacuum (see Figure 9). The Roborock S5 has a suite of sensors including ultrasonic ranging, collision bump sensors, IR cliff sensors, and, most importantly, a Light Detection and Ranging (LIDAR) sensor that allows the robot to implement Simultaneous Localization And Mapping (SLAM) to build detailed maps of the environment for the purpose of increasing cleaning efficiency. The robot has two major processors (https://github.com/dgiese/dustcloud-documentation/tree/master/roborock.vacuum.s5 (accessed on 30 March 2021).): an Allwinner R-16 with four ARM A7 CPU cores and an STM32 microcontroller. The device runs Ubuntu with Player 3.10-svn, which provides the mapping functionality [37,38].

**Figure 10:**
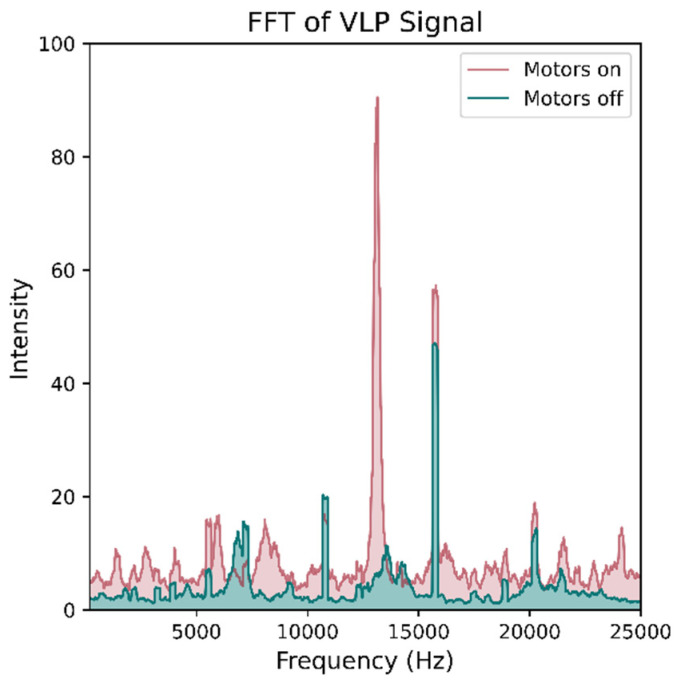
Visualization of noise caused by the EMI of the vacuum’s motors. The green plot shows a DFT of the VLP signal when the motors are off and luminaires are operating. The distinct peaks represent signals detected from the luminaires in proximity. The red shows the DFT of the received signal at the same location but with the vacuum motors on. In addition to a large peak at around 12.5 kHz, the background noise level has increased over the entire frequency range.

The vacuuming and brush electric motors could not be removed without the operating system disabling the vacuum entirely. Therefore, to reduce power consumption, the vacuum was modified by removing the motor driving gears. Upon restart, the robot has an initialization routine of around 5 s, during which time the motors wind up. This places a limit on the collection frequency of the system. If stationary readings were not required, then the robot would not need to be stopped. A small delay is also needed between start and stop Application Programming Interface (API) commands of around 0.5 s to prevent the system from temporary locking messages. The robot vacuum’s motors can introduce noise into the VLP system as shown in Figure 10. If readings were to be taken while the robot is still moving, some form of electromagnetic shielding would be required. However, during this experiment, the robot was fully stopped before taking each reading, meaning this was not required.

The open-source home automation software platform, Home Assistant (https://github.com/home-assistant (accessed on 30 March 2021)), was used to control the area where VLP measurements were made. It has integrated control for the Roborock S5 vacuum and provides a Python package exposing the robot’s API calls. Before using the API package, a token for the device must be extracted to authenticate commands (https://www.home-assistant.io/integrations/xiaomi_miio/ (accessed on 30 March 2021)). Once set up, the API provides a convenient way to issue numerous commands, which include starting/stopping of automated cleaning, forward/backward/rotation commands, and moving to specific co-ordinates (in the robot maps reference frame). The Python API calls can also be used in conjunction with the Xiaomi Home mobile app (https://play.google.com/store/apps/details?id=com.xiaomi.smarthome&hl=en_NZ&gl=US (accessed on 30 March 2021)), which allows for easy setup configuration. After placing the robot in a new location and starting a cleaning routine, the will builds a map of the environment and display it along with the position of the robot.

**Figure 11:**
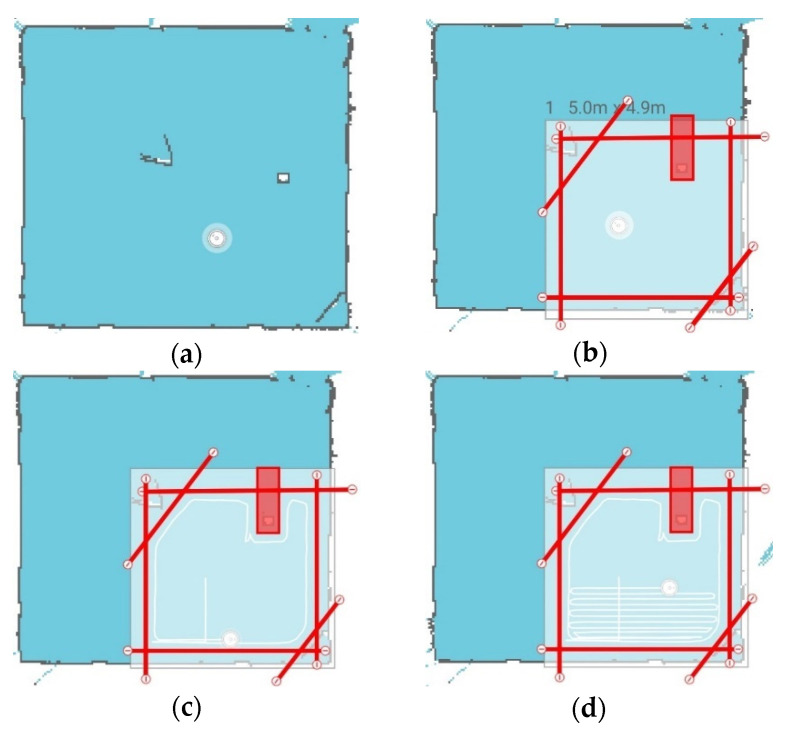
Setting up the cleaning zone using the Xiaomi Home mobile app. When placed in a new environment, the vacuum will build a map of its environment as shown in (**a**); the triangle in the rooms’ center is the cardboard deflectors placed on the Vive base stations stands (Figure 1). Within the app, walls and zones can be placed that will restrict the movement of the robot (**b**). Once initialized, the robot will then traverse the perimeter (**c**) before sweeping back and forth to cover the entire area (**d**).

As shown in Figure 11, after placing “virtual walls” (in red), a “cleaning zone” can be defined (light color), and the robot will be restricted in its movement. Once the “cleaning routine” begins, the robot travels around the perimeter before sweeping back and forth over the entire area. The API commands can pause the operation to allow for fingerprinting measurements to be captured and then resume “cleaning” without disrupting progress.

The utility of the map stems from the ability to define “no-go” regions within the app that limit the operating range of vacuum. This allows control of where the robot will focus its data collection when performing fingerprinting and provides easy segmentation of an environment. The robot can also avoid colliding with walls, pillars, and other objects within the environment which reduces the possibility of damaging attached sensors and avoids wasted time as the robot would otherwise use the bump sensor and continually ‘bounce’ off walls.

## 3. RSS Data

### 3.1. Experimental Setup and Data Collection

**Figure 12:**
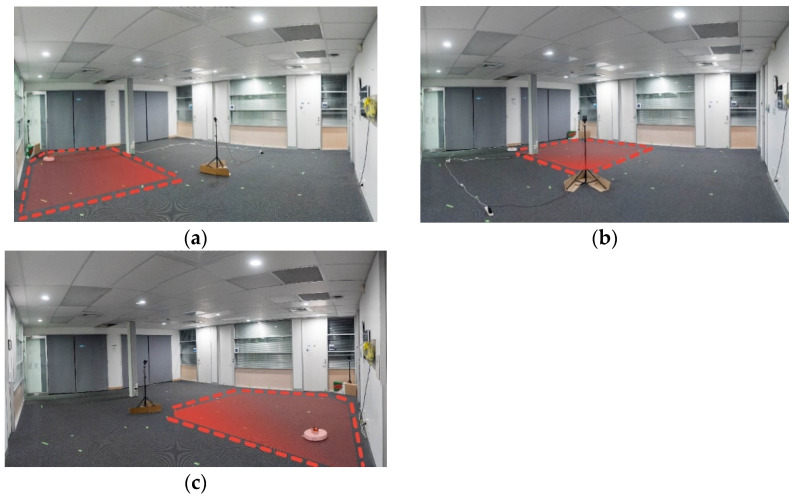
The area of the lobby was too large to be covered by the Vive 1.0 system, which necessitated splitting the environment into 4 quadrants. Each quadrant required a separate transformation to align the Vive readings with the rooms co-ordinate reference frame. Three quadrants are shown in (**a**–**c**) respectively.

**Figure 13:**
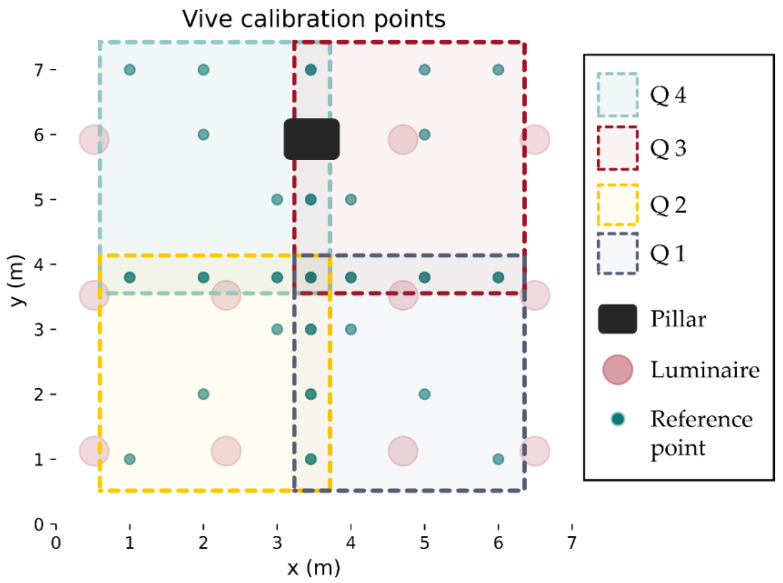
Calibration points used for deriving the transformations. Twenty-six reference points were carefully mapped out using a laser level and tape measure in such a way that each quadrant would have 10 reference calibration points, sharing four with each neighbor. This reduced the overall number of calibration points required.

The HTC Vive 1.0 version that was used in this work was unable to track the robot over the entire 6.5 m × 7 m floor area of the lobby. The newer v2.0 (https://www.vive.com/eu/accessory/base-station2/ (accessed on 30 March 2021)) version of these base stations allows for tracking with 4 stations covering 10 m × 10 m (with the possibility of this increasing further in the future (https://www.roadtovr.com/htc-experiments-with-16-base-stations-steamvr-tracking-2-0-multi-room/ (accessed on 30 March 2021))), which would cover the entire lobby area and provide more line-of-sight links in environments with obstacles. To collect ground truth with the v1.0 lighthouses, the room was split into four quadrants, taking measurements at each quarter (Figure 12 shows the area of three quadrants) and then merging the data set. This process could be repeated indefinitely, allowing for Vive ground truth tracking of an arbitrary large area. Reference locations for calibration were placed on the lobby floor via manual measurements with the aid of a laser level line. The reference points were selected to provide common points between quadrants to reduce the required number of points (see Figure 13). The investigation into the number of required reference points shown in Figure 8b demonstrated that at least 5 were needed. However, 10 points were used for each quadrant to safeguard from erroneous readings. Before taking VLP measurements in each quadrant, the Vive lighthouses were set up and the tracker was positioned on each of the 10 reference positions, and measurements were taken. These recorded points were then used to define the translation from the Vive co-ordinate frame to the room frame using the process outlined in Section 2.3. The Roborock S5 was configured using the mobile app to sweep the quadrant avoiding walls and the Vive base stations. A Python script running on desktop connected to the local network was used for data collection and robot control. The script would call an API to pause the vacuum, make an HTTP request to the VLP receiver for a new reading, and then call the Vive API to get the ground truth position. The data were timestamped and written to a csv file. The cleaning routine was then resumed with another API call. While moving, the change in position of the robot was measured by continuous polling of the Vive tracker, and once it moved a certain threshold distance (180 mm), the robot would pause to take another reading. This method was found to perform much better than a simple time delay due to the robot taking more time at edges, where it must turn around, than in the middle of a long straight run.

### 3.2. VLP Data Preprocessing

Data collected from the four quadrants were merged into one set, and the VLP readings were processed to extract the RSS values from each of the 11 luminaires. The experiment collected VLP data and Vive ground truth at 7477 different locations. Outliers in the data set were identified using the *z* component of the Vive tracker position. Values that deviated from the tracker’s height (which remained constant) indicated that the tracker had made erroneous position estimates. This could have been caused by line-of-sight (LOS) disruptions, network traffic congestion, or the IMU dead reckoning used by the Vive malfunctioning. Positions where the *z* estimate differed from the overall mean by more than 3 standard deviations were discarded, which equated to 133 points (or 1.8%). Therefore, the final data set contains RSS values at 7344 measurement locations.

**Figure 14:**
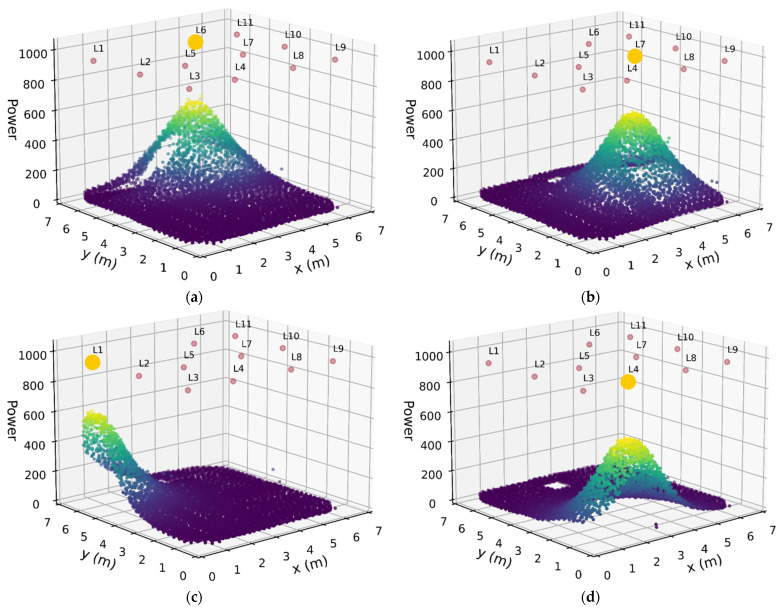
RSS plots for luminaires 6 7, 1, and 4 in (**a**), (**b**), (**c**) and (**d**) respectively. The signal is largest when the sensor is directly beneath and falls off as distance increase. No data were collected at the location of the pilar which can be seen clearly in (**d**).

The collected data set can be visualized by plotting the RSS for a particular luminaire at each fingerprint location. Figure 14 shows plots for a selection of luminaires. As expected, the highest RSS values were beneath a luminaire and falling off with distance.

**Figure 15:**
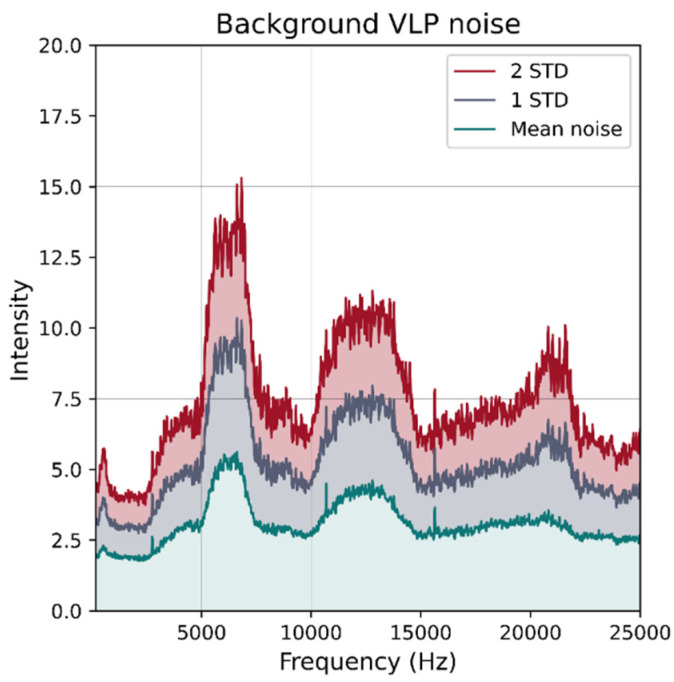
Background VLP noise with no active luminaires. One hundred eighty DFTs were computed at 10 s intervals and for each frequency the average was calculated and plotted (green) 1 and 2 standard deviations above the mean level were also calculated to provide an indication of the noise floor.

The photodiode used in the receiver has a half angle of 60 degrees, which limits the maximum range at which it can reliably detect signals from the luminaires. Furthermore, beyond a certain distance, the VLP signal falls below the level of the noise floor. To obtain an approximate measure of the noise floor, the receiver was placed at a fixed position in the room and the VLP luminaires were powered for illumination without injecting the modulation signal. One hundred eighty readings were taken at intervals of 10 s. Figure 15 shows the average noise level over all frequencies of interest as well as the 1 and 2 standard deviations above. From this figure, it can be seen that the noise is generally below “15” and used as a threshold to clamp the RSS readings to 15 (if they are below the threshold).

To the best of the authors’ knowledge, this data set is the largest reported VLP data collection. The final labeled data set is publicly available and has been posted on GitHub (https://github.com/tyrel-glass/Public-VLP-Data set (accessed on 30 March 2021)).

### 3.3. Lambertian Channel Model Calibration

**Figure 16:**
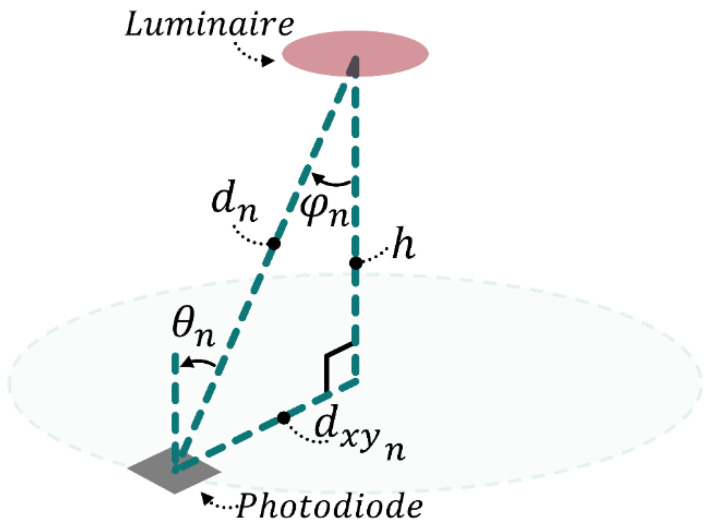
When both the luminaire and receiver are horizontal, $\theta_{n}$ and $\varphi_{n}$ are equal, and $d_{n}$ then forms the hypotenuse of a right-angle triangle.

The Lambertian model that relates RSS to the distance of the target from the luminaire (and several other parameters) can be expressed as [39]:

(6) $$P_{r_{n}}=\frac{P_{t_{n}}}{d_{n}^{2}}\left(\frac{m_{n}+1}{2\pi }\right)\cos^{m_{n}}(\varphi_{n})Acos\left(\theta_{n}\right)$$

here, $P_{r_{n}}$ is the received power at the target location at a distance $d_{n}$ away from luminaire *n*, $P_{t_{n}}$ is the transmitted power of that luminaire, $m_{n}$ is the Lambertian order, $\varphi_{n}$ and $\theta_{n}$ are the irradiance and incidence angles, respectively, and A is the photodiode area. Please see Figure 16 for an illustration of some of the channel parameters.

$P_{r_{n}}$ is extracted following the process described in Section 2.1. Since the luminaires and the photodiode are parallel for the experimental setup, Equation (6) simplifies to

(7) $$P_{r_{n}}=P_{r_{n},0}{\left(\frac{d_{n,0}}{d_{n}}\right)}^{m_{n}+3}$$

here, $P_{r_{n},0}$ is the RSS at a chosen reference location, which is $d_{n,0}$ distance away from the *n*th luminaire. Model calibration is performed by obtaining the values of the three parameters: $m_{n}$, $P_{r_{n},0}$, and $d_{n,0}$.

$P_{r_{n},0}$ and $d_{n,0}$ are derived by averaging the RSS and the distance of three closest points to the nth luminaire. Once $P_{r_{n},0}$ and $d_{n,0}$ have been determined for each luminaire, Equation (7) can be rearranged to determine [17]

(8) $$m_{n}=\frac{\log\left(\frac{P_{r_{n}}}{P_{r_{n},0}}\right)}{\log\left(\frac{d_{n,0}}{d_{n}}\right)}-3$$

$m_{n}$ can then be estimated at a set number of data points and then averaged.

**Figure 17:**
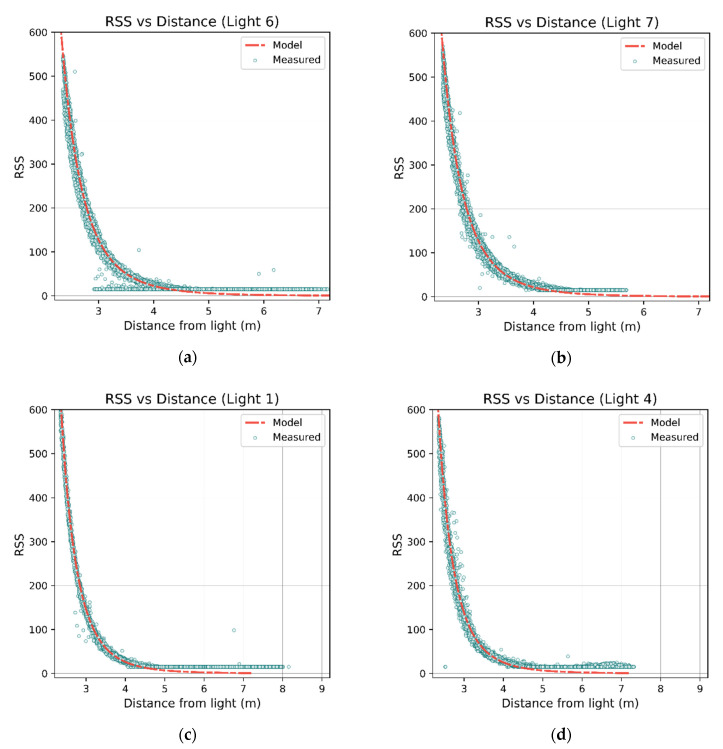
RSS vs. distance plots for luminaires 6, 7, 1, and 4 in (**a**–**d**) respectively. The red lines show the trained Lambertian models. Luminaire 6 in (a) shows clear deviation due to the presence of the pillar; some effects can be seen in luminaires 1 and 7 (in (**b**,**c**)), although not as severe. Luminaire 7 is located close to the center of the room and therefore does not have as wide a range of distances compared to the others. Clamping of the RSS values can also be clearly seen in all plots, as the distance increases and readings do not follow the Lambertian model (which approaches zero).

Figure 17 shows the distance vs. RSS scatterplot for a selection of luminaires with the calibrated model overlaid in red. Of note is Luminaire 6, which displays clear deviation due to its close proximity to a pillar.

## 4. Machine Learning Models for Positioning

To demonstrate the utility of a dense fingerprint, several positioning algorithms were implemented on the data set and the effect of varying volumes of training data investigated. Positioning was framed as a multi-target supervised regression problem,$\;\hat{y}=f\left(X\right)$**,** where both the x,y co-ordinates of the receiver $\hat{y}=\left(\hat{x},\hat{y}\right)$ were predicted using the RSS values from 11 luminaires at each position i.e., $X=\left(RSS_{1},\;RSS_{2},\;\ldots ,RSS_{11}\right)$. The machine learning models implemented were polynomial regression, polynomial ridge regression [40], Support Vector Regression (SVR) [41], Random Forest (RF) [42], Weighted k Nearest Neighbour (k-NN) [43], and Multilayer perceptron (MLP) [44]. These models were selected as they are some of the most common Machine Learning algorithms used for regression [45]. Algorithms were implemented using scikit-learn version 0.24.1 (https://scikit-learn.org (accessed on 30 March 2021)). SVR does not support multitarget output, so two regressors were trained, one for each of the x and y components of the co-ordinates.

**Figure 18:**
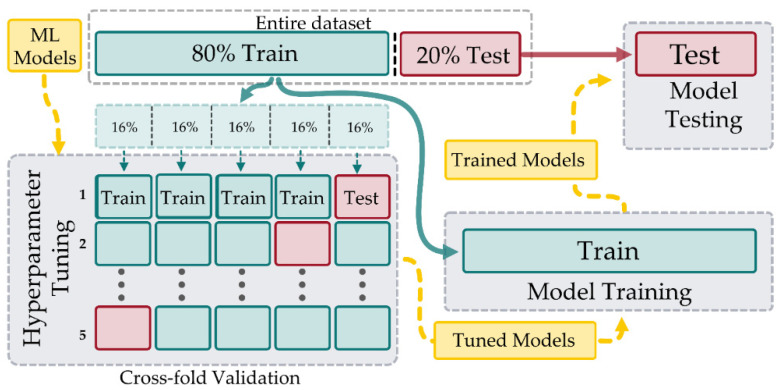
The data set is initially split into 80% training and 20% testing. The ML hyperparameters are then tuned using 5-fold cross-validation of the 80% training data by splitting into 5 folds and iterating 5 times through the folds, each time selecting 4 folds for training and the remaining for validation. The resulting five scores are then averaged to compute the score for a set of hyperparameters. Once tuned, the ML models are retrained using all of the 80% training data and assessed for final performance on the 20% test set.

Each ML model has various hyperparameters that need to be specified, and these can have a large impact on performance. To make fair comparisons between models, the hyperparameters were tuned using fingerprint data. Table 3 lists the hyperparameters requiring tuning for each model. The data set was partitioned using an 80–20 train–test split. The training data was used for hyperparameter tuning, as well as for retraining the tuned models. The tuned models were then validated against the 20% test set to make the final performance assessment. The overall process for training and testing is illustrated in Figure 18.

The method of tuning comprised two successive rounds of grid-search, where the hyperparameter values were swept through a set range. For the first sweep, progressive powers of 10 were used ($\ldots 10^{-2},\;10^{-1},10^{0},\;10^{1},\;10^{2}\ldots$) as a range for each parameter and every combination of values was tested. Testing of a set of hyperparameters was undertaken using 5-fold cross-validation on 80% of the training data. The training set was split into five distinct subsets with the model being trained and evaluated five times, selecting a different fold for evaluation every time and training on the remaining four. The 5 evaluation scores were then averaged to provide an overall evaluation. Mean Square Error (MSE) was the performance metric used for selecting the best parameters. If the best performing parameters were at the upper or lower range of values, the range was extended to ensure the optimum values were contained within the search range. This process was then repeated with powers of 2 ($2^{-1},\;2^{0},\;2^{1},\;2^{2}$), with the best performing set providing the final hyperparameters for each model. The choice of cross-fold validation rather than a train/validation split was to avoid hyperparameter overtraining by constantly evaluating on a smaller subset of the data. The tuned parameters and the cross-validation score for each model are presented in Table 3.

**Figure 19:**
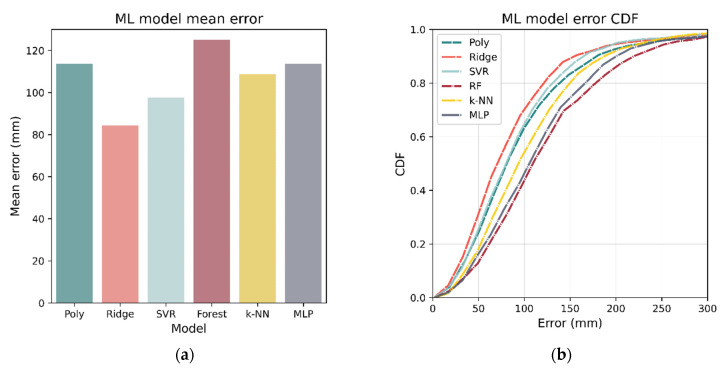
Mean and CDF for errors of tuned models trained on the entire 80% training set. Errors were evaluated using the 20% test set, and it can be seen, in both (**a**,**b**), that ridge regression has the best performance, with RF being the worst.

After the optimum hyperparameters were determined, each model was then re-trained on the entire 80% training set (instead of cross-fold validation) and tested against the 20% test set. The localization accuracies of the various models on the 20% test set are listed in Table 4. Using all metrics (mean, median, RMSE), ridge regression was the best performing model with a mean of 84.4 mm. Figure 19 shows the mean error as well as the CDF for each of the models.

## 5. Channel Model Based Positioning

Unlike the ML-based localization presented in the previous section, these methods rely on modeling the physical characteristics of the systems. The two methods presented both involve ranging—estimating distance from a luminaire—followed by computing a position from multiple distance estimates to fixed points using lateration.

### 5.1. Ranging

The RSS-distance model calibrated in Section 3.3 can be used to estimate the distance of the target to a luminaire from an RSS reading:

(9) $$d_{n}=d_{n,0}{\left(\frac{p_{r_{n},0}}{P_{r_{n}}}\right)}^{\frac{1}{m_{n}+3}}$$

**Figure 20:**
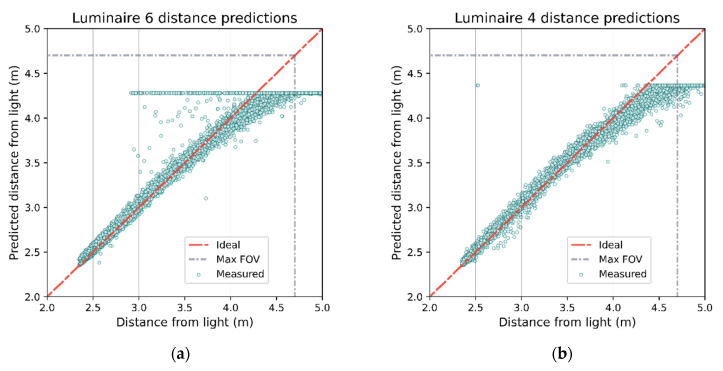
Actual distances to luminaire vs. distances expected based on RSS model for luminaires 4 and 6. Both luminaires demonstrate linear behavior up until the photodiodes begin to reach their FOV (grey dashed line). The pillar near luminaire 6 causes a cluster of points that clearly violate the model (**a**) where the predicted distance is greater than the actual distance. The predicted distance for each luminaire will be limited due to the clamping of RSS (an example for luminaire 4 in (**b**)).

The estimated ranges can then be compared against the ground truth recorded distances (see Figure 20). As the distance from a luminaire increases, the accuracy of the estimate tends to degrade as indicated by increasing spread.

### 5.2. Positioning

Following ranging, two different algorithms were implemented for position estimation: lateration using linear least squares [46] and spring relaxation [13].

#### 5.2.1. Linear Least Squares

The least-squares method involves determining the intersection of several circles centered at the x-y location of each luminaire (which was precisely measured) with radii given by the estimated radial distance determined by ranging. A receiver at a distance $d_{XY_{n}}$ (calculated by $\sqrt{d_{n}^{2}-h^{2}}$ using Figure 16—h being the ceiling height) from luminaire n at location $x_{i},y_{i}$ will lie on circle with the equation given by

(10) $$d_{XY_{n}}{}^{2}={\left(x-x_{i}\right)}^{2}+{\left(y-y_{i}\right)}^{2}$$

For a localization system with N luminaires, there will exist N such equations. Expansion, subtraction, and rearrangement of these equations leads to the following [46]:

(11) $$[\begin{array}{cc} x_{2}-x_{1} & y_{2}-y_{1}\\ x_{3}-x_{1} & y_{3}-y_{1}\\ \vdots & \vdots \\ x_{N}-x_{1} & y_{N}-y_{1} \end{array}][\begin{array}{c} x\\ y \end{array}]=\frac{1}{2}\left[\begin{array}{c} x_{2}^{2}-x_{1}^{2}+y_{2}^{2}-y_{1}^{2}+d_{XY_{1}}^{2}-d_{XY_{2}}^{2}\\ x_{3}^{2}-x_{1}^{2}+y_{3}^{2}-y_{1}^{2}+d_{XY_{1}}^{2}-d_{XY_{3}}^{2}\\ \vdots \\ x_{N}^{2}-x_{1}^{2}+y_{N}^{2}-y_{1}^{2}+d_{XY_{1}}^{2}-d_{XY_{N}}^{2} \end{array}\right]$$

which is of the form Ax=b**,** where $\left(x_{1},y_{1}\right),\left(x_{2},y_{2}\right)\ldots \;\left(x_{N},y_{N}\right)$ are the locations of the luminaires 1, 2…N and $d_{XY_{1}},\;d_{XY_{2}},\;\ldots ,d_{XY_{N}}$ the estimated distances based on ranging. Given inherent signal and system noise, the circles will not overlap perfectly, and a least-squares approximation can be used:

(12) $$\bar{x}={\left(A^{T}A\right)}^{-1}A^{T}b$$

#### 5.2.2. Spring Relaxation

Spring relaxation utilizes the same ranging process based on the Lambertian model in Equation (6) but estimates the final position via an iterative process that simulates hypothetical springs connecting the estimated position with each N luminaire [13]. The relaxed length of each spring is the estimated ranging distance given by Equation (9). At each possible x,y location, a force can be calculated based on the extension/compression of each spring. Beginning with an initial location estimate given as the mean position of the N luminaires with highest RSS readings, the algorithm iteratively refines the position by moving a small step in the net force generated by the hypothetical springs. After each new position the forces are then recalculated, and the next step direction is determined. The algorithm is run until it converges on a solution, determined by the next step being below a set threshold.

**Figure 21:**
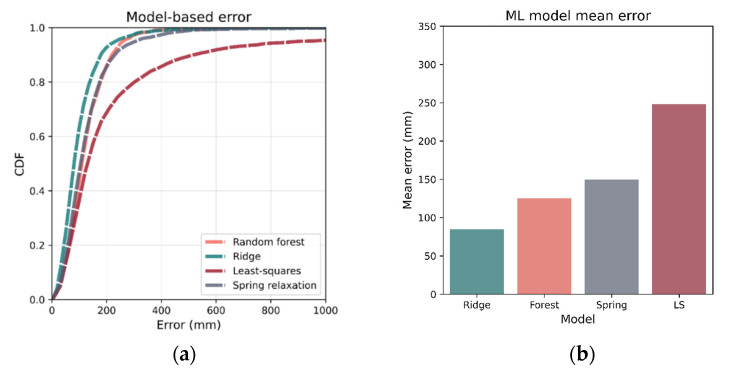
CDF and mean of localization errors for channel model-based positioning are benchmarked in (**a**,**b**) respectively. Spring relaxation was the better performing of the model-based techniques, although its performance was slightly lower than that of RF—the worst-performing ML model. The least-squares method was considerably worse than any of the other methods. The models were trained/calibrated on the entire 80% training set and tested on the 20% test set.

To determine N, the optimum number of luminaires to use for lateration or the spring relaxation method, 5-fold cross validation was again implemented using the 80% training set. For both methods, it was found that N=4 was the best number of luminaires. The channel models were then calibrated using the entire training set and validated against the test set. Figure 21 gives the mean errors as well as CDF for both methods along with Ridge regression, the best-performing ML positioning technique, for comparison. It can be observed that among the two lateration techniques, the spring relaxation-based method is more accurate (also seen in Table 5). However, both are outperformed by the Ridge regression-based positioning.

## 6. Importance of Data Size

### 6.1. Training

**Figure 22:**
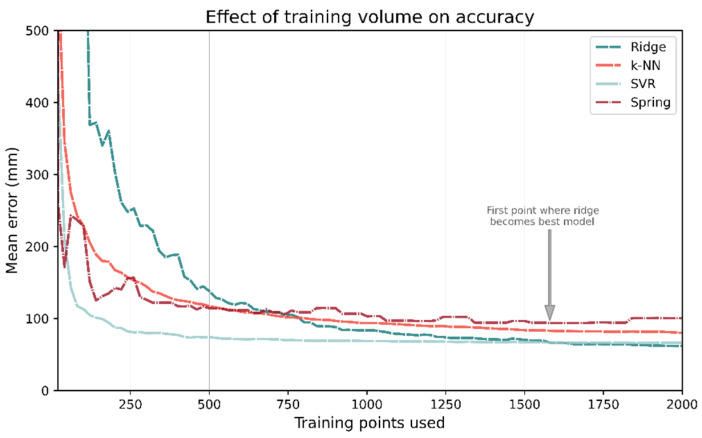
The number of training points used has a large impact on which model has the best performance. Despite being the best performing model when trained on the entire data set, Ridge regression requires a significant number of training data. It takes 1580 training points before Ridge regression becomes the best performing model.

To investigate each ML- and channel-based model’s dependence on the size of training data, the models were repeatedly trained or calibrated on progressively larger fractions of the training data after they had been randomly shuffled. Each model was trained on 20 training samples and its performance evaluated on the test set; this was then repeated for 40, 60, etc. The plots for the number of training points vs. mean test accuracy are shown in Figure 22. If the training data are restricted to just 100 samples, SVR outperforms all other methods. In fact, for training points 45–1560, SVR in general has the lowest mean error. As more training data are used, ridge regression becomes the best performing model; however, it takes 1580 training points for this to occur.

**Figure 23:**
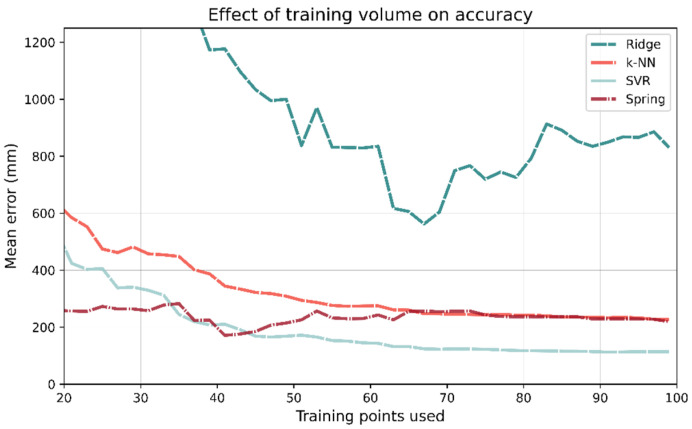
If few training points are used (<33), then the spring relaxation model-based approach outperforms the ML methods.

Figure 23 shows the same results from the same analysis but using a step size of 2 with a maximum 100 training points. With 31 training points, the mean accuracy of spring relaxation is 258 mm. The same is true for ridge regression is 1562 mm, almost an order of magnitude greater.

### 6.2. Testing

**Figure 24:**
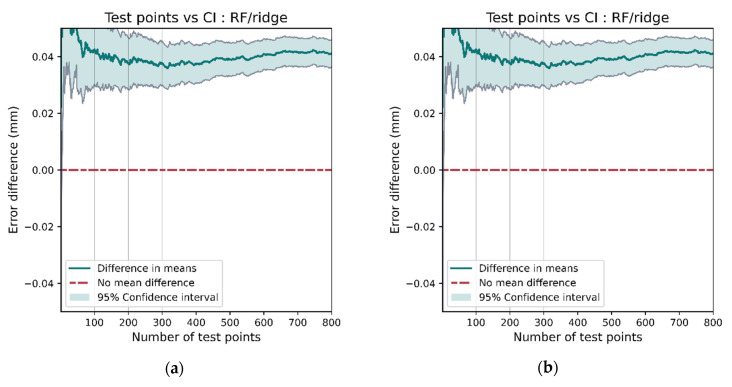
There is a clear difference in the performance of the RF and ridge models, shown by 0 not being contained in the 95% confidence interval (**a**) and the *p*-value being clearly below the 0.05 significance level (**b**).

Having a large data set allows for robust comparisons between models as the confidence in each model’s accuracy can be ascertained using hypothesis testing. As an example, we can compare the performance between ridge regression and random forest using a paired *t*-test on the errors for each model at test positions. The null hypothesis, assuming no true mean difference between the performance between the models, would need to be rejected before we can confidently conclude a model’s superiority. Figure 24a shows the 95% confidence interval for the difference in the positioning error and Figure 24b displays the *p*-value for the *t*-test. Since the two models were the best and worst performing, the results are as expected. Note that 0 is not included within the 95% confidence interval and the *p*-value is well below the 0.05 threshold. This rejects the hypothesis that there is no difference in model performance.

**Figure 25:**
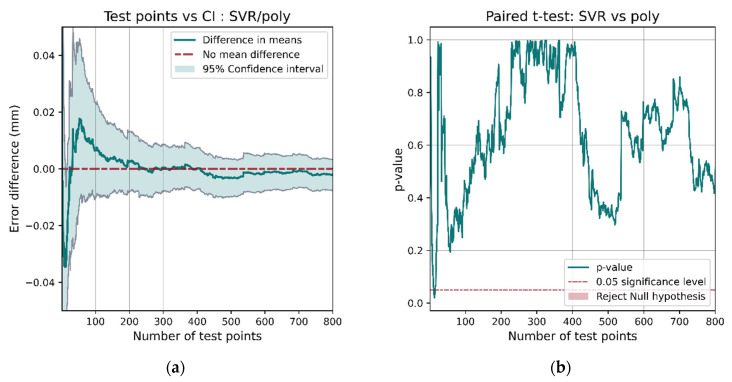
When comparing SVR and polynomial regression, the performance of the models is more similar. The confidence interval (**a**) includes 0 a for a large proportion of the data, and the *p*-value (**b**) only drops below the 0.05 significance level for a brief period with very few samples.

If we instead compared models whose performances are more similar such as SVR and polynomial regression, with SVR having better performance on the entire test set (Table 4), the results are not as conclusive. The confidence interval for the performance interval (Figure 25a) contains 0 most of the time. The *t*-test *p*-value only dips below the 0.05 significance value for a short interval and then increases again. This casts doubt on the superiority of SVR over polynomial regression.

**Figure 26:**
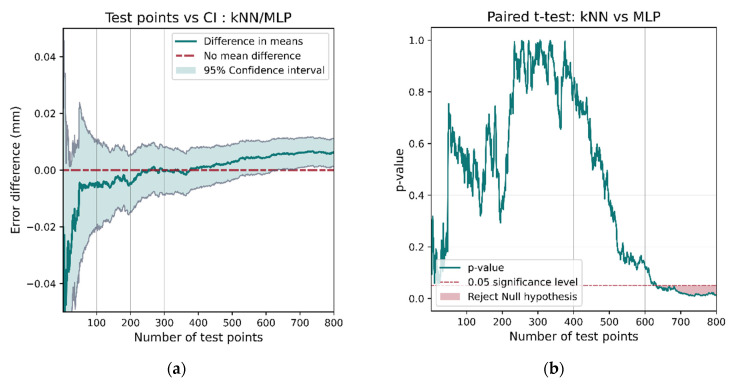
The *t*-test between the k-NN and MLP models does indicate a performance difference but only after more than 600 test points have been assessed. This demonstrates the need for a large data set when making robust comparisons between models. If less than 600 points had been used for testing, then the confidence interval (**a**) would include 0, the *p*-value (**b**) would be larger than 0.05, and the hypothesis that there was no difference in performance would not be rejected.

The comparison of k-NN and MLP show that they exhibit different behavior. k-NN is the better performing model (as shown in Table 4); however, a confidence interval for the mean difference in model performance (Figure 26a) includes 0 until around 600 test points are assessed. This results in the *t*-test failing to reject the hypothesis that there is no difference in model performance until 600 test points. This demonstrates that a large data set is necessary not only for training models but also for robust performance comparisons.

## 7. Conclusions and Future Work

An autonomous data collection method for collecting RSS data for a VLP system was developed. This is the first such system reported in the literature to allow the automated collection of a large VLP data set over a wide area. The experimentally collected RSS data set is an order of magnitude larger than other reported data sets. It has been made available online for other researchers. Examples of performance analyses of different VLP techniques using this data set were presented. Memoryless positioning techniques using both ML- and model-based ranging and lateration were investigated. Ridge regression was found to be the most accurate method for localization achieving mean and 90th percentile errors of 84.4 and 144 mm. However, if a small data set is available (33<), model-based positioning using spring relaxation performs most accurately with a mean of 258 mm.

It should be noted that RSS was used as the feature for the ML algorithms. Feature generation—deriving new variables from the raw RSS values, such as powers and interactions [47] or RSS ratios [48]—can improve localization accuracy. It is also possible to train multiple models on subsets of the luminaires, then aggregate the predictions [49] or frame the positioning as a classification problem [50]. These investigations provide scope for future research. Furthermore, decoupling the x,y prediction into two single-target methods results in independent predictions and the relationships between the variables are lost and cannot be exploited. Analysis of this problem is beyond the scope of this paper but may be worthwhile as only limited cases of decoupled models have been investigated [50,51]. A separate, yet similar issue is the method of loss function used to train multi-target regressors. ML positioning models are often trained using one-dimensional loss functions. However, since positioning deals with two-dimensional data, the model should be trained to minimize the Euclidean error of the positioning system. Future research can investigate the utilization of 2D custom loss function for ML training. The analysis presented in this paper was restricted to data collected at a constant height and with obstacles removed from the test area. Future work could investigate how 3D positioning or changing environments affect the findings presented here.

## Figures and Tables

**Table 1:** VLP articles listed by size of experimental data set. Most articles use a limited number of data points and do not describe how ground truth was recorded.

Research	Number of Data Points	Year	Testbed Size (mxm)	Mean Accuracy (mm)	Method	Ground Truth
Alam et al. [17]	693	2018	3.3 × 2.1	27	WKNN	Laser rangefinder
Guo et al. [18]	225	2019	0.7 × 0.7	50	KNN, RF, ELM	Not stated
Vongkulbhisal et al. [19]	160	2012	1.8 × 1.2	148	KNN	Not stated
Zhang et al. [20]	100	2019	1.8 × 1.8	34	NN	Not stated
Zhang et al. [21]	100	2019	1.8 × 1.8	18.8	NN, MMBP	Not stated
Chuang et al. [22]	81	2019	0.5 × 0.5	40	Regression	Not stated
Wu et al. [23]	81	2020	0.5 × 0.5	30	Regression	Not stated

**Table 2:** Variation in Vive measurement in mm for a stationary target. Each run was conducted with a different tracker position and orientation.

Run	Mean	Median	90th Percentile	Max
A	0.138	0.115	0.249	0.460
B	0.137	0.109	0.263	0.642

**Table 3:** Results of the hyperparameter tuning. The listed parameters for each of the models were tuned using two rounds, the first being successive powers of 10, the second being powers of 2. The scoring metric for grid search was RMSE.

Model	Parameters		Cross Validation Score RMSE (mm)
Polynomial regression	Degree:	4	109
Ridge regression	Degree:	5	97.4
	Alpha:	0.05	
SVR	Kernel:	poly	130
	Epsilon:	0.05	
	Degree:	3	
	Coef0:	1	
	C:	10	
RF	Estimators:	200	120
	Max depth:	10	
	Min samples split:	2	
	Min samples leaf:	1	
	Max leaf nodes:	200	
k-NN	k-neighbors:	5	114
	Weights:	Distance	
	Distance Metric:	Manhattan	
MLP	Layers:	5	109
	Nodes/layer:	100	
	Alpha (L_2_ penalty):	10^−5^	

**Table 4:** Accuracy of the ML in mm after being retrained on the entire 80% training set and tested on the 20% test set.

Model	Median	Mean	RMSE	90th Percentile
Polynomial regression	76.3	114	395	172
Ridge regression	68.9	84.4	133	144
SVR	82.0	97.6	143	165
RF	109	125	166	220
k-NN	94.1	109	151	190
MLP	99.9	114	156	197

**Table 5:** Accuracy of the model-based methods after being retrained on the entire 80% training set and tested on the 20% test set. The RMSE for both is greater than the 90th percentile errors, indicating that there are some large errors. All values are in mm.

Model	Median	Mean	RMSE	90th Percentile
Spring relaxation	106	150	292	258
Least squares	130	248	450	590

## Data Availability

The VLP data collected is available for download at https://github.com/tyrel-glass/Public-VLP-Dataset (accessed on 30 March 2021). For data citation, please cite this article.
